# Validated HPLC-RI Method for the Determination of Lactulose and its Process Related Impurities in Syrup

**DOI:** 10.4103/0250-474X.65027

**Published:** 2010

**Authors:** A. Nelofar, A.H. Laghari, A. Yasmin

**Affiliations:** Pakistan Council of Scientific and Industrial Research, Laboratories Complex, Karachi-75280, Pakistan

**Keywords:** Lactulose, syrup formulation, HPLC-RI, method development

## Abstract

A simple, swift with good sensitivity and reproducibility, HPLC-RI method has been developed for the quantification of lactulose and related compounds (fructose, galactose, epilactose and lactose) in oral suspension formulation. The analysis was carried out by using mobile phase (water and acetonitrile 75:25) at the flow rate of 1.0 ml/min. on isocratic HPLC-RI system. After manipulating mobile phase composition and mobile phase flow rate a good separation of five components was achieved within 15 minutes of run time. This study is beneficial to determine the active ingredient as well as related compounds simultaneously, without using buffer in mobile phase which causes bad resolution and has limitation to analyze on other hyphenated techniques such as LC-MS.

Lactulose is a semi-synthetic disaccharide made from lactose by a chemical reaction which was first described in 1930[1]. Lactulose is formed by the LA transformation of lactose via a 1, 2-enediol intermediate. Lactulose is much less stable in solution than lactose and may subsequently degrade via β-elimination to give galactose, tagatose and saccharinic acids and other low molecular weight products[2]. According to United States Pharmacopeia (2008) specification lactulose syrup must not contain more than 16% galactose, 12% lactose, 8% epilactose and 1% fructose. Consequently a reliable method is needed to determine process related impurities in lactulose syrup.

Several methods for determination of lactulose in different samples are cited[3,4] in literature. However, when we tried to follow USP method we didn‘t achieve good resolution; it also used buffer in mobile phase and has longer retention time. Now it is the first time that we are going to report a method to quantify lactulose and process related sugar compounds in syrup formulation with a simple mobile phase composition and within a short time span.

Reference standard of lactulose was obtained from Platinum Pharma, Karachi, Pakistan. Acetonitrile HPLC grade (Fischer Scientific UK Limited, Bishop Meadow Road, Lough Borough, Leicestershire LE 11 5RG UK) and de-ionized water (filtered) were used to prepare the mobile phase. Standard fructose, galactose, and lactose were of E. Merck AG, Darmstadt, Germany, while epilactose was of USP grade. Commercial samples of lactulose syrup were purchased from local market of Karachi, Pakistan.

The liquid chromatographic system consisted of Perkins Elmer Series 200 equipped with gradient pump, column oven and Refractive Index detector. Analysis was conducted on an amino column (3 µNH_2_ 4.6×150 mm) while maintaining the temperature of column oven at 40°. The mobile phase was consisted of acetonitrile-water (75:25 % v/v), filtered through a 0.45 µm Millipore filter and degassed in an ultrasonic bath before delivering into the system. The samples were introduced through an injector valve having a 20 µl sample loop, while maintaining the flow rate of 1 ml/min using isocratic pump system. Detector has adjustable attenuator range from 0.25 to 512×10^-6^RIU. Best results were achieved at 256×10^-6^RIU, so it was chosen for analysis. Response was set at standard and polarity at positive, see Table 1.

**TABLE 1:** OPTIMISED CHROMATOGRAPHIC CONDITIONS

HPLC			R-I (Detector)		
Mobile phase	Flow rate	Column oven temperature	Range	Response	Polarity
Acetonitrile:water (75:25)	1.0 ml/min (isocratic flow)	40°	256	Standard	Positive

Standard stock solutions of 100 ppm of individual lactulose, fructose, galactose, epilactose and lactose in acetonitrile and water (1:1) were prepared in separate volumetric flasks. Working solutions were prepared by diluting the stock solutions with the same solvent to contain (0.125-10 µg/ml).

The viscous drug syrup was shaken thoroughly to make homogenous mixture. A quantity of the syrup equivalent to label claim of lactulose was transferred accurately into a 100-ml calibrated volumetric flask containing acetonitrile and water mixture (1:1). The content of the flask was shaken for about 10 min and diluted to volume with the same solvent. The solution was then filtered through a 0.45-µm Millipore filter. Desired concentration for the drug was obtained by accurate dilution and the analysis was followed up as in the general analytical procedure.

The method was validated for selectivity, linearity, precision, accuracy, and extraction recovery by observing the inter-day and intra-day, data accumulated by running standards and samples respectively. Selectivity was evaluated by comparing the chromatograms obtained by running five samples of blank as well as drug spiked with higher and lower concentrations of analytes. The calibration curves of all standards were constructed at five concentrations in the range of 1-8 µg/ml. Data are shown in Table 2. The intra-day precision and accuracy were evaluated by determining replicate analysis of standards and samples on the same day. For inter-day same practice was done on different days. Extraction recovery and matrix effects were determined by running five replicates of all samples by varying extracting solvents, concentrations of spiked analytes and extraction time. Final calculations were based on peak area response obtained from all studies.

**TABLE 2:** DETERMINATION OF LACTULOSE AND PROCESS RELATED IMPURITIES IN SYRUP

Compound	Concentration (mg/ml)		RSD (%)	
	Added	Found (mean±SD)	Intra-day	Inter-day
Fructose	1	0.98±0.083	2.42	7.10
	2	1.99±0.096	3.16	8.20
	8	8.20±0.246	4.17	10.11
Galactose	1	1.10±0.042	3.11	8.12
	2	1.96±0.031	4.21	9.20
	8	7.90±0.193	6.62	10.01
Epilactose	1	0.99±0.072	2.54	5.12
	2	1.98±0.062	3.12	7.81
	8	7.78±0.210	4.81	8.21
Lactose	1	1.11±0.036	2.32	3.91
	2	2.04±0.061	2.51	4.02
	8	8.14±0.182	4.10	6.10
Lactulose	1	1.08±0.048	2.89	3.21
	2	1.98±0.031	3.81	6.29
	8	8.02±2.208	6.91	9.30

SD is standard deviation and RSD is relative standard deviation. Intra-day, n = 5; inter-day, n = 3 series per day for 3 days

Optimization of mobile phase is crucial for improving various chromatographic parameters; peak shapes, detection, sensitivity and retention time. Aqueous acetonitrile in various proportions having 10 to 30% water were used for analysis and the best chromatographic separation was achieved with acetonitrile water (75:25 v/v). Representative chromatogram is shown in fig. 1.

**Fig. 1:**
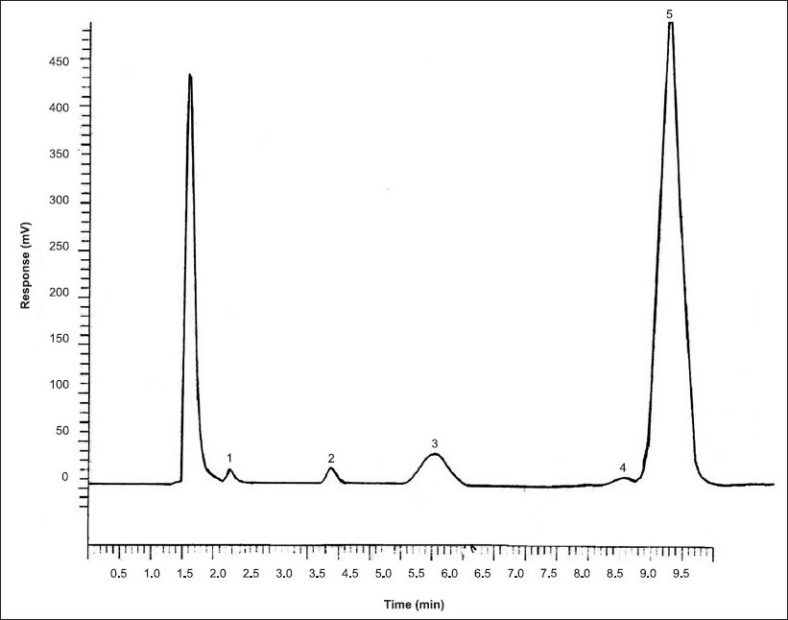
Chromtogram of sugars The chromatographed sugars were 1. fructose, 2. galactose, 3. epilactose, 4. lactose, 5. lactulose

The HPLC system was equilibrated with the initial mobile phase composition, followed by 5 injections of the same standard. These 5 consecutive injections were used to evaluate the system suitability on each day of method validation. The system suitability parameters include capacity factor, resolution and asymmetric factor. All parameters were found satisfactory with good specificity for the stability assessment of lactulose, fructose, galactose, epilactose and lactose

Selectivity was determined by comparing the chromatograms of blank samples with the corresponding spiked samples. No interference from endogenous substance was observed at the retention time of lactulose, fructose, galactose, epilactose and lactose. Carry-over was eliminated by rinsing system; this was confirmed by analyzing blank samples immediately following the samples at highest concentration.

Linearity was tested by assaying drug sample, spiked with known volumes of reference standard stock solution at concentrations 0.125-10 µg/ml. Injected concentration versus recovered concentration were plotted and the correlation coefficients were calculated.

The precision of the method was investigated with respect to repeatability. For intra-day precision, 5 samples of seven concentrations were analyzed on the same day. Data are summarized in Table 3. Generally acceptable repeatability of the results within one day and day-to-day was observed.

**TABLE 3:** VALIDATION AND SYSTEM SUITABILITY

Compound	Linearity range (mg/ml)	Calibration equation*	Correlation factor (R^2^)
Fructose	1-8	y= 272128x+24169	0.9989
Galactose	1-8	y= 31193x+2513	0.9989
Epilactose	1-8	y= 320318x+28700	0.9989
Lactose	1-8	y= 345354x−130250	0.9909
Lactoluse	1-8	y= 6E+06x+580698	0.9989

* Five data points with a sample size of n= 3 for each

The limit of detection (LOD) and limit of quantitation (LOQ) of this method were determined from the coefficient of variation of a known concentration of reference standards. The LOD for this assay, calculated from three times the noise level of the response, is 0.0625 μg/ml. The LOQ for this assay calculated from ten times the noise level of the response, is 0.125 µg/ml.

In this study, a HPLC-RI method has been developed that offers a feasible and reliable means of measuring lactulose in pharmaceutical dosage formulation. A fully validated and efficient HPLC-RI procedure for the determination and quantification of lactulose and related compounds is developed, without using buffer for the first time to optimize sensitivity and accuracy. Validation statistics showed that this method possesses good sensitivity, precision, and repeatability. Linearity was confirmed over a wide calibration range with regression coefficients higher than 0.9901. The assay method was applied to a drug formulation and the successful detection of five sugars showed that this method is suitable for detecting and determining amounts of lactulose fructose, galactose, epilactose and lactose (Table 4). Hence, this method can be recommended for the routine quality control of this drug.

**TABLE 4:** DETERMINATION OF PROCESS RELATED IMPURITIES IN DRUG SAMPLES

Drug Sample	Fructose (%)	Galactose (%)	Epilactose (%)	Lactose (%)
Floralic	0.8	9.3	2.6	5.7
EZ-fl ow	0.9	10.2	2.1	4.9
Dphalec	0.7	10.6	1.9	5.7

USP (2008) recommendation; fructose, galactose, epilactose and lactose should not be more than 1 %, 16 %, 8 % and 12 %, respectively in lactoluse syrup
